# Trends and factors associated to early initiation of breastfeeding, exclusive breastfeeding and duration of breastfeeding in Ethiopia: evidence from the Ethiopia Demographic and Health Survey 2016

**DOI:** 10.1186/s13006-019-0248-3

**Published:** 2020-01-09

**Authors:** Berhanu Teshome Woldeamanuel

**Affiliations:** Department of Statistics, College of Natural Sciences, Salale University, Fitche, Oromia Ethiopia

**Keywords:** Breastfeeding, Early initiation, Exclusive breastfeeding, Trends, Duration of breastfeeding, Ethiopia, Demographic Health Survey

## Abstract

**Background:**

Initiation of breastfeeding immediately after birth, exclusive breastfeeding, and continuous breastfeeding for at least 2 years lower the risk of newborn deaths. This study was conducted to examine the trends and factors associated with early initiation of breastfeeding, exclusive breastfeeding and duration of breastfeeding in Ethiopia.

**Methods:**

Data for this study were extracted from the Ethiopian Demographic and Health Survey 2016. A total of 5122 children were included in the analysis. Multivariate logistic regression analysis, and Cox proportional hazards model were fitted to find the factors associated with breastfeeding practices. Reported *p* - values < 0.05 or a 95% Confidence Interval of Odds Ratio/Hazard Ratio excluding one was considered as significant association with early initiation of breastfeeding, exclusive breastfeeding, duration of breastfeeding and independent variables.

**Results:**

About 81.8% of the children initiated breastfeeding within 1 h of birth and during the day before an interview, 47% were exclusively breastfed during the first 6 months. The median duration of breastfeeding was 22 months (22 ± 0.50 months 95% Confidence Interval [CI] 21.01–22.99). Rural residents (Odds Ratio [OR] 0.71, 95% CI 0.51, 0.99), mothers with no antenatal follow up (OR 0.75, 95% CI 0.57, 0.99), caesarean birth (OR 0.80, 95% CI 0.66, 0.96) and home delivery were associated with low initiation of breastfeeding within 1 h of birth. Mothers with no/primary education (OR 0.62, 95% CI 0.40, 0.96), no baby postnatal checkup (OR 0.53, 95% CI 0.39, 0.73), average/larger size of a child at birth (OR 0.80, 95% CI 0.65, 0.99) and deliveries outside of health centers were significantly associated with non-exclusive breastfeeding at the time of the interview. Further, mothers living in Amhara (Hazard Ratio [HR] 1.31, 95% CI 1.05, 1.64), Oromia (HR 1.27, 95% CI 1.04, 1.54), and Benishangul-Gumuz (HR 1.34, 95% CI 1.09, 1.65) regions had a longer duration of breastfeeding while Muslims, employed mothers, multiple births and poor economic level of households were associated with shorter durations of breastfeeding.

**Conclusions:**

Rural residence, female sex, home delivery, caesarean birth, small birthweight baby and large family size were associated with late initiation of breastfeeding. Living in Affar, Somali, and Harari, primary education level of mothers, giving birth outside of health facilities, no antenatal care follow up, and no postnatal check-up were associated with non-exclusive breastfeeding, while younger age mothers, Muslims, giving birth outside of health facilities, and employed mothers were associated with shorter time to cessation of breastfeeding. Providing health education and counseling for mothers during and after pregnancy should be encouraged.

## Background

Globally, about 800,000 neonatal deaths are attributed to late initiation of breastfeeding and lack of exclusive breastfeeding [1]. Initiation of breastfeeding immediately after birth can reduce the risk of neonatal mortality in the first week of life by 22% globally [2]. Furthermore, exclusive breastfeeding of infants is strongly associated with a lower risk of post neonatal death [3, 4].

By the year 2030, the Sustainable Development Goals (SDG) target reducing neonatal deaths to 12 per 1000 live births, and under-five deaths to less than 25 per 1000 live births through eliminating preventable child deaths [5]. Sub-Saharan African (SSA) countries have the highest neonatal mortality rate, about 28 deaths per 100 live births annually. Ethiopia is one of the SSA countries with a high rate of neonatal and infant mortality. The latest Ethiopian Demographic and Health Survey [6] reported that 92% of children born 5 years preceding the survey, initiated breastfeeding within the first hour after birth, and 58% were exclusively breastfed the day before an interview. Under-five mortality has been declining in Ethiopia, where the majority of deaths occur in the neonatal and infant period caused by lack of important nutrients and by infections [6]. Initiating breastfeeding in the first 1 h after birth can decrease the risk of newborn infant mortality by about 45% and exclusively breastfed children were 14 times more likely to survive the first 6 months of life than non-breastfed children [7]. Under nutrition is the main causes of child mortality, and UNICEF 2010 estimated about 40 to 60% of under-five stunting were subjected to late initiation and non-exclusive breastfeeding [8].

Despite early initiation of breastfeeding, the majority of children do not breastfeed exclusively in the countries of Africa [9]. The World Health Organization (WHO) recommends early newborn breastfeeding initiation within 1 h of birth, exclusive breastfeeding for 6 months, and continued breastfeeding for 2 years or more in conjunction with complementary foods [10–13].

Breast milk has many benefits of ensuring a healthy child and the survival of a child. Several studies reported that the initiation of breastfeeding within a day was significantly associated with reducing low birthweight related neonatal mortality and infection related neonatal mortality among all live births [14–17]. Worldwide, 10% of the disease burden in under-five children were due to the non-exclusive breastfeeding. According to The Lancet, estimate suboptimal breastfeeding is a consequence of 1.4 million child deaths and 77% of child deaths are accounted for by non-exclusive breastfeeding in the first 6 months of birth [18]. The highest risk of inappropriate feeding during the first 6 months of life occurs in developing countries where 96% of all infant mortality is due to suboptimal breastfeeding [19]. Nutritional deficits among infants are immediate consequences of delayed breastfeeding and non-exclusive breastfeeding that leads to morbidity and mortality among children [20].

Breastfeeding prevalence is high in Ethiopia. However, the practice of exclusive breastfeeding among 0–6 months age children differs by regions. According to Ethiopian Demographic and Health Survey (DHS) 2016 nearly all children, i.e., 97% are breastfed at some points while only 58% of infants below the age of 6 months are exclusively breastfed. Among Ethiopian regions, the level of early initiation of breastfeeding and the median duration of exclusive breastfeeding are minimal in Affar region, 43% and 2.7 months, respectively, and median duration of predominant breastfeeding among children born 5 years preceding the survey were minimal for Somali region (3.8 months). In addition to breast milk, only 7% of children 6–23 months old receive the least acceptable dietary standards while only 14% of children had a sufficiently diverse diet [6]. In Ethiopia, the Ministry of Health (MoH) established the National Nutrition Program II (NNP II) and the National Guideline on Adolescent, Maternal, Infant, and the Young Child Nutrition initative 2016 targeted promoting optimal feeding and care practices, encouraging mothers to exclusively breastfeed their child for the first 6 months without any additional fluids or foods and continuing breastfeeding up to a child is 2 years old. Nearly three-fourth (73%) of children began breastfeeding within 1 hour of a birth and 92% within a day of birth. Eight percent of children received prelacteal feeding. In Affar region the percentage of prelacteal feeding was 41%.

According to various studies, and WHO/UNICEF report, the factors associated with breastfeeding practice differ with socioeconomic, demographic, behavioral and cultural factors of mothers, place and mode of delivery, professional counseling on breastfeeding, and obstetric and health service related factors [21–23]. Identifying factors associated with breastfeeding practices are essential to decrease the neonatal and infant death rates due to preventable causes from lack of necessary nutrients. Despite the fact that a number of studies have been done investigating the factors associated with early initiation time, exclusive breastfeeding and duration of breastfeeding in Ethiopia, breastfeeding practice is still less than optimal and more effort is needed. Thus, this study tries to assess the major risk factors of delayed initiation, exclusive breastfeeding and duration of breastfeeding, with consideration to various demographic, socioeconomic, and health service related factors, based on 2016 Ethiopia Demographic and Health Survey data. It may help to evaluate the Sustainable Development Goals (SDGs), aimed at reducing under-five death rates of 25 deaths per 1000 live births through increasing optimal feeding patterns among infants, and to avoid preventable childhood deaths by 2030 [24].

## Methods

### Study setting

The data for this study were extracted from the Ethiopian Demographic and Health Survey (EDHS) 2016 [6, 25]. The Central Statistical Agency (CSA) together with the Ministry of Health (MoH) and the Ethiopian Public Health Institute conducted the survey from January 18, 2016 - June 27, 2016 and The United States Agency for International Development (USAID) funded the survey. The nationwide survey has information on a range of socioeconomic and demographic factors of the population. It implemented a two-stage sampling within the nine regions and two administrative cities of a country. In the first stage, 645 enumeration areas (202 in urban areas and 443 in rural areas) were selected with probability proportion to size. The second stage involved selection of 28 households per cluster of an equal probability of being included in the systematic selection of the newly formed household list. The EDHS 2016 has three questionnaires: the household questionnaire, the woman’s questionnaire and the man’s questionnaire. All women of 15–49 years old, who were either a permanent inhabitant or visitors who lived at least one night in the household before the survey, were eligible for the interview. Data were gathered by conducting face-to-face interviews with women that met the eligibility criteria.

### Data and study population

**Fig. 1:**
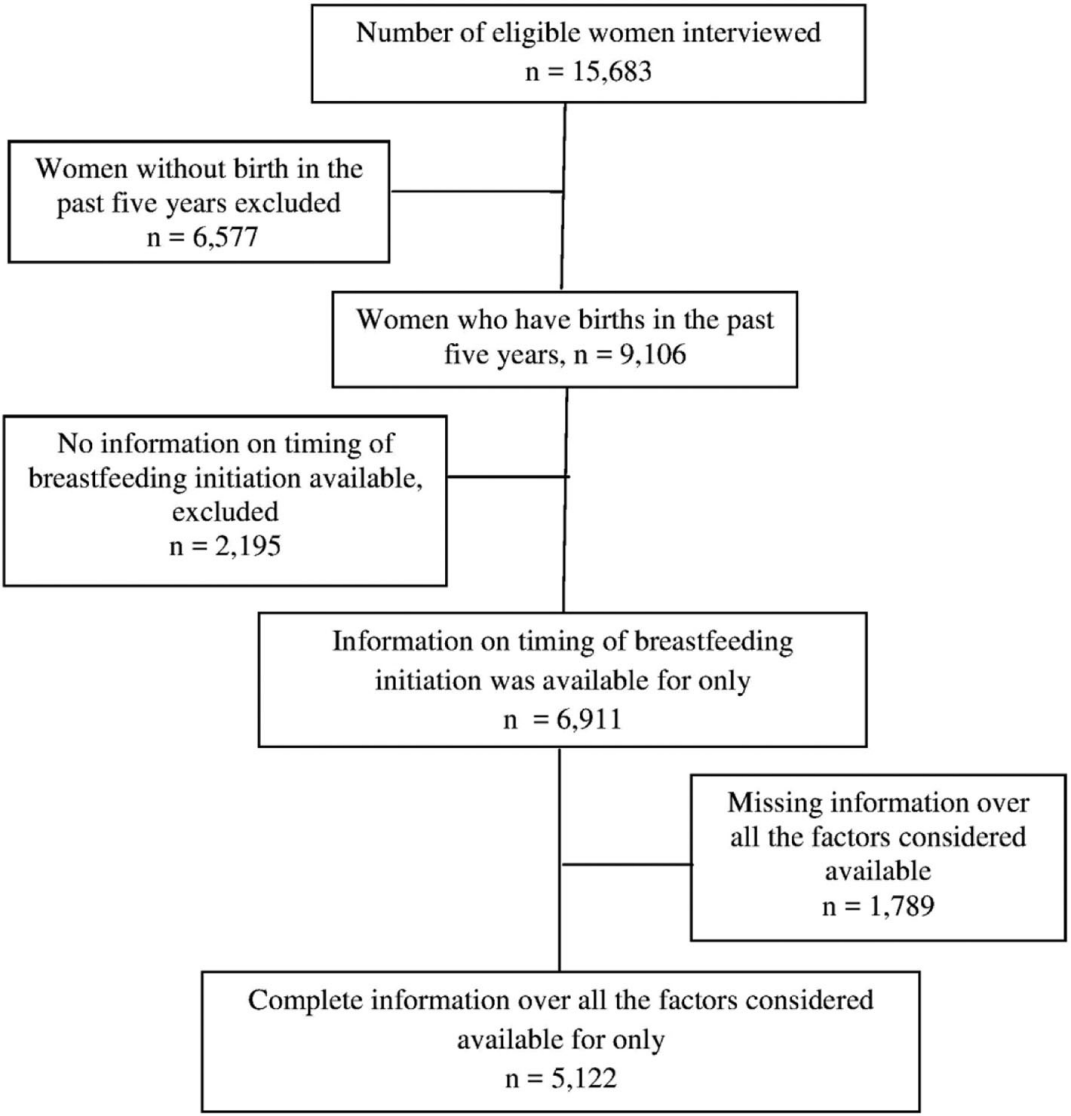
Diagrammatic presentation of data extraction from the 2016 Ethiopian Demographic and Health Survey

A total of 15,683 eligible women were interviewed in 2016 EDHS. About 5122 children with complete information on timing of breastfeeding initiation, and born 5 years preceding the survey were identified. In the case of more than one child per household, in this study we used data collected only from the last birth. The details of data extraction procedures are presented in Fig. 1.

### Variables

This study has three dependent variables: early initiation of breastfeeding (binary outcome categorized as 1 if a mother initiated breastfeeding in the first hour after birth and 0 otherwise), exclusive breastfeeding (binary outcome categorized as 1 if a mother did not feed the baby anything else excepts of syrups and medicines apart from breast milk on the day before the interview during the first 6 months after birth and 0 otherwise), and the duration of breastfeeding the last child of the respondent (calculated as the number of months that the mother reports having breastfed her baby). The data on breastfeeding practices and associated factors were taken from the woman’s questionnaire.

The main independent variables were mother’s education, father’s education attainment, maternal age, wealth index of household, religion, place of residence, region of residence, professional antenatal and postnatal care, place of delivery, mode of delivery, type of birth, sex of child, size of child at birth, employment status of mother and parity was selected from the available similar studies on the subject [26–34]. The DHS use five wealth quintiles. Households are given scores based on the number and kinds of consumer goods they own, ranging from a television to a bicycle or car, in addition to housing characteristics such as source of drinking water, toilet facilities, and flooring materials. These scores are derived using principal component analysis. National wealth quintiles are compiled by assigning the household score to each usual (de jure) household member, ranking each person in the household population by her or his score, and then dividing the distribution into five equal categories, each comprising 20% of the population.

### Data analysis

Descriptive characteristics of the subjects were presented as frequencies and percentages to summarize the distribution of selected background characteristics of mothers and children. To examine the socioeconomic and demographic factors associated with early initiation and exclusive breastfeeding (odds ratios with their 95% confidence intervals), logistic regression analysis was performed using Stata statistical package version 13 [35]. Further, a Cox’s Proportional Hazards model was employed to examine factors associated with time to cessation of breastfeeding. Multicollinearity between covariates was checked using the variance inflation factor (VIF) and the goodness of fit of the fitted models was checked using the likelihood ratio test (LRT).

## Results

### Descriptive statistics of breastfeeding practice by some characteristics

The median duration of breastfeeding was 22 ± 0.50 months (95% CI 21.01–22.99), while that of mean duration was 28.92 ± 0.32 months (95% CI 28.29–29.56). Table 1 shows that the percentage of early initiation of breastfeeding was lower in the Affar region (54.7%) followed by the Amhara (75.1%) and the Tigray regions (78.9%), respectively, while the highest percentage was observed in Dire Dawa (95.4%) and Harari (93.7%) regions.

**Table 1:** Background characteristics of mothers and children, EDHS 2011 (*N* = 5122)

Background characteristics/Covariates	Categories	N (%)	Early initiation of breastfeeding in the 1st hour		Exclusive breastfeeding for 6 months	
			Early initiation (%)	Delayed (%)	Yes (%)	No (%)
Region	Tigray	497 (9.7)	78.9	21.1	48	52
	Affar	459 (9)	54.7	45.3	30.5	71.5
	Amhara	578 (11.3)	75.1	24.9	58.8	41.2
	Oromiya	771 (15.1)	88.6	11.4	51.2	48.8
	Somali	620 (12.1)	83.7	16.3	27.7	72.3
	Benishangul-Gumuz	449 (8.8)	81.3	18.7	53.7	46.3
	SNNPR	684 (13.4)	87.9	12.1	53.2	46.8
	Gambela	339 (6.6)	83.2	16.8	57.2	42.8
	Harari	285 (5.6)	93.7	6.3	39.3	60.7
	Addis Ababa	200 (3.9)	84	16	49	51
	Dire Dawa	240 (4.7)	95.4	4.6	49.6	50.4
Place of Residence	Rural	4278 (83.5)	80.6	19.4	47.2	52.7
	Urban	844 (16.5)	87.8	12.2	46.7	53.3
Maternal age	≤ 24	754 (14.7%)	81.8	18.2	37	63
	25–29	1522 (29.7)	83.2	16.8	44.3	65.7
	30–34	1306 (25.5)	82.3	17.7	45.6	54.4
	35–39	985 (19.2)	80.7	19.3	54.2	45.8
	40 and higher	555 (10.8)	78.9	21.1	59.8	40.2
Husband education	No education	3520 (68.7)	80.3	19.7	46.6	53.4
	Primary	1187 (23.2)	84.2	15.8	48.4	61.6
	Secondary / Higher	415 (8.1)	88	12	48.2	51.8
Mother education	No education	2675 (52.2)	78.4	21.6	47	53
	Primary	1645 (32.1)	85.9	14.1	48.3	51.7
	Secondary	450 (8.8)	83.1	16.9	43.3	56.7
	Higher	352 (6.9)	87.2	12.8	47.7	52.3
Religion	Coptic orthodox	1561 (30.5)	79.4	20.6	53.4	46.6
	Protestant	951 (18.6)	85.6	14.4	54.4	45.6
	Muslim	2486 (48.5)	81.5	18.5	40.3	59.7
	Traditional/Others	124 (2.4)	88.7	11.3	49.2	50.8
Wealth index	Poorest	1820 (35.5)	75.3	24.7	41.2	49.6
	Poorer	901 (17.6)	83.5	16.5	52.9	47.1
	Middle	769 (15)	85.7	14.3	51.9	49.1
	Richer	679 (13.3)	84.5	15.5	51.3	48.7
	Richest	953 (18.6)	87.6	12.4	49.4	50.6
Sex of child	Male	2616 (51.1)	80.3	19.6	47.5	52.5
	Female	2506 (48.9)	83.4	16.6	46.8	53.2
Place of delivery	Home	3345 (65.3)	79.4	20.6	47.4	52.6
	Health	1777 (34.7)	86.3	13.7	46.6	53.4
Baby postnatal checkup	No	4686 (91.5)	82	18	47.1	52.9
	Yes	436 (8.5)	79.8	20.2	47.7	52.3
Antenatal visits	0	1927 (37.6)	79.7	20.3	47	53
	1–3	1509 (26.5)	81.5	18.5	45.7	54.3
	4+	1686 (32.9)	84.5	15.5	48.6	51.4
Mode of delivery	Normal	4992 (97.5)	82.5	27.5	47.2	52.8
	Caesarian	130 (2.5)	56.2	43.8	43.8	56.2
Type of birth	Single birth	5035 (98.3)	82	18	47.1	52.9
	Multiple birth	87 (1.7)	73.6	26.4	48.3	51.7
Size of child at birth	Large (> 4 kg)	1560 (30.5)	83.6	16.4	49.4	50.6
	Average (2.5-4 kg)	2186 (42.7)	83.7	16.3	47.6	52.4
	Small (< 2.5 kg)	1376 (26.9)	76.9	23.1	43.9	56.1
Employment status	Not working	3669 (71.6)	80.3	19.7	45.8	54.2
	Working	1453 (28.4)	83.7	16.3	50.6	49.3
Parity	1–2	1061 (20.7)	84.9	15.1	45.9	54.1
	3–4	1687 (32.9)	82.2	17.8	47	53
	5–6	1225 (23.9)	81.2	18.8	48.9	51.1
	7+	1149 (22.4)	79	21	46.6	53.4
Total			81.8	18.2	47.1	52.9

The percentage of exclusive breastfeeding was low in the Somali region (27.7%) and the Affar region (30.5%). More than three-quarters (83.5%) of the mothers were from rural areas. About 80.6% of rural newborns initiate breastfeeding within 1 h of birth compared to 87.8% of urban newborns. The percentage exclusive breastfeeding was almost uniform in both rural and urban residents, 47.2 and 46.7%, respectively.

More than two-thirds (68.7%) of the mothers had no formal education at all. The percentage of early initiating of breastfeeding (80.3%) and exclusive breastfeeding (46.6%) was lowest among uneducated mothers. Similarly, more than half (52.2%) of fathers were illiterate. Nearly half (48.9%) of the children included were females. Male babies had a higher proportion of exclusive breastfeeding (47.5%) than that of females. Table 1 also shows that 65.3% of the mothers delivered outside of health facilities. Babies born outside of health facilities had a smaller percentage (79.4%) of early initiation of breastfeeding.

Only 8.5% of babies had a postnatal check-up. And 62.4% of mothers had attended antenatal care at least once. About 71.6% of mothers were unemployed. More than half (53.1%) were from a poor economic level. Moreover, children born from unemployed women and poor wealth indices had a lower percentage of early initiation of breastfeeding and exclusive breastfeeding practices 80.3 and 75.3%, respectively. Similarly, children with a small birthweight, cesarean birth, and being born of a large family (7 or higher), had a lower percentage of early initiation of breastfeeding, i.e. 79, 76.9 and 56.2%, respectively (Table 1).

**Fig. 2:**
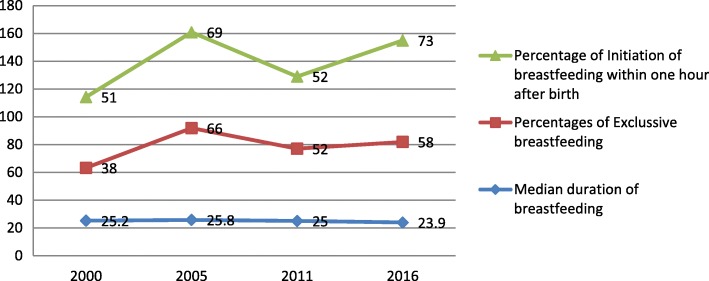
Trends of breastfeeding practice in Ethiopia

**Fig. 3:**
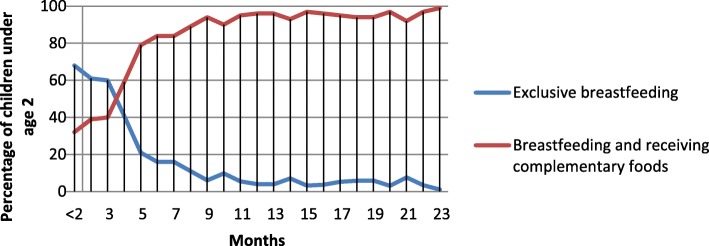
Breastfeeding practice by age of child

Trends of breastfeeding practices are presented in Figs. 2 and 3. The percentage of early initiation of breastfeeding within increases from 51% in 2000 to 69% in 2005, then it fell to 52% after 5 years in 2011 and rose to 73% in 2016. Similarly, percentages of exclusive breastfeeding increase significantly from 38% in 2000 to 58% in 2016. Both initiations of breastfeeding and exclusive breastfeeding show a decreasing trend from 2005 to 2011. Overall, this shows that the percentages of both early initiations of breastfeeding and exclusive breastfeeding increased from 2000 to 2016. The median duration of breastfeeding in Ethiopia decreased from 25.20 months in 2000 to 23.90 months in 2016. See Fig. 2.

The two outstanding stages, three and 5 months in Fig. 3 indicate that the period when a child terminated exclusive breastfeeding.

**Fig. 4:**
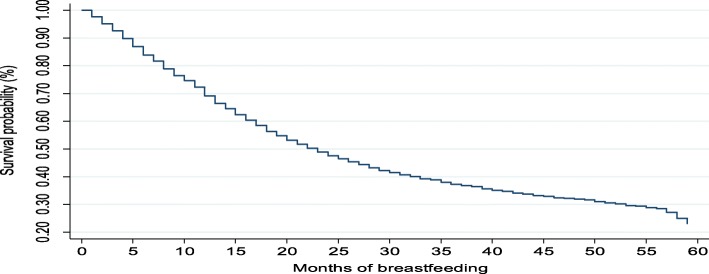
Kaplan-Meier survival function for time to cessation of breastfeeding

**Fig. 5:**
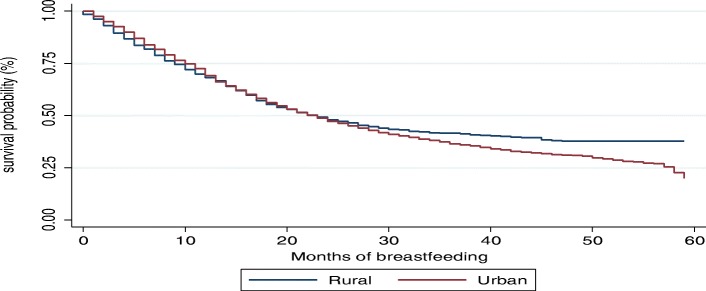
Kaplan-Meier survival function by place of residence

**Fig. 6:**
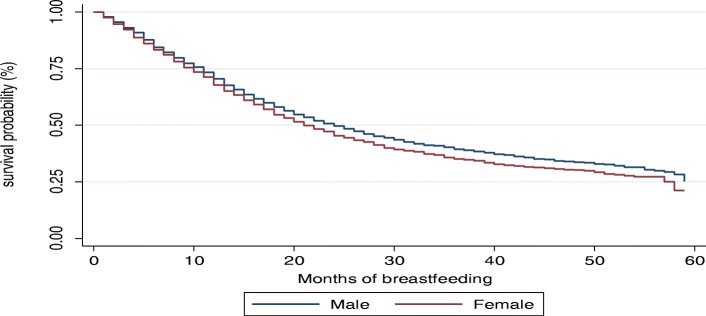
Kaplan-Meier survival function by sex of child

**Fig. 7:**
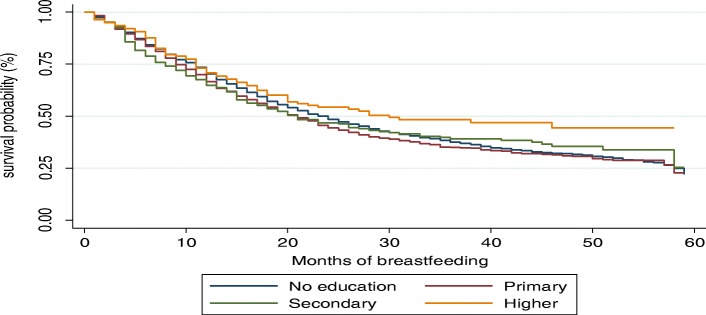
Kaplan-Meier survival function by mother education

The Kaplan-Meier survival functions presented in Figs. 4, 5, 6 and 7 indicate a consistently falling time to cessation of breastfeeding. The Kaplan-Meier survival function of rural women on Fig. 5 was above that of urban women, indicating that, on average, rural women breastfed for a longer time compared to urban women. Similarly, the survival function of females on Fig. 6 lies below that of male children. On the other hand, Fig. 7 shows mothers with secondary or higher education level have a longer time to cessation of breastfeeding, i.e., educated mothers breastfed for a longer time compared to that of illiterate mothers.

### Factors associated with early initiation of breastfeeding

Multivariate logistic regression analysis to examine factors associated with early initiation of breastfeeding is presented in Table 2. The goodness of fit was also checked using the likelihood ratio tests (LRT). Consequently, the result of likelihood ratio test, provided 1521.32 (*p* - value < 0.0001), would imply good fit for the model. Thus, the null hypothesis, there is no difference between the model with no predictor variables and the model with explanatory variables was rejected. Accordingly, regions of residence, place of residence, religion of mother, sex of a child, place of delivery, mode of delivery, birthweight of a child, baby postnatal checkup and parity, were covariates significantly correlated with early initiation of breastfeeding.

**Table 2:** Factors associated with initiation of breastfeeding and exclusive breastfeeding, EDHS 2011 (*N* = 5122)

Background characteristics/Covariates	Categories	Early initiation of breastfeeding in the 1st hour		Exclusive breastfeeding for 6 months	
		OR	95% CI for OR	OR	95% CI for OR
Region (Dire Dawa)	Tigray	4.71	2.32, 9.53	0.460	0.23, 0.92
	Affar	13.81	7.11, 26.81	5.972	3.53, 10.11
	Amhara	5.30	2.65, 10.59	0.524	0.27, 1.00
	Oromiya	2.40	1.22, 4.71	0.390	0.21, 0 .72
	Somali	3.63	1.86, 7.08	6.442	3.85, 10.78
	Benish.Gumuz	4.32	2.18, 8.53	0.404	0.21, 0 .79
	SNNPR	2.50	1.24, 5.04	0.818	0.43, 1.54
	Gambela	3.78	1.80, 7.90	0.913	0.45, 1.85
	Harari	1.19	0.54, 2.64	3.563	2.04, 6.23
	Addis Ababa	3.66	1.64, 8.17	2.062	1.03, 4.14
Residence (Rural)	Urban	0.71	0.51, 0.99	0.802	0.57, 1.13
Maternal age (40+)	≤ 24	1.14	0.77, 1.68	1.101	0.71, 1.70
	25–29	1.01	0.74, 1.37	1.046	0.73, 1.50
	30–34	1.04	0.78, 1.38	1.412	1.02, 1.96
	35–39	1.04	0.79, 1.37	1.095	0.79, 1.52
Husband education (Secondary / Higher)	No education	1.42	0.90, 2.24	1.289	0.81, 2.06
	Primary	1.52	0.98, 2.36	1.206	0.77, 1.90
Mother education (Higher)	No education	1.06	0.68, 1.63	0.781	0.51, 1.20
	Primary	0.91	0.59, 1.40	0.622	0.40, 0.96
	Secondary	1.24	0.79, 1.93	0.878	0.56, 1.38
Religion (Traditional /Others)	Coptic orthodox	1.89	1.02, 3.50	1.836	0.86, 3.92
	Protestant	1.62	0.88, 2.97	0.956	0.45, 2.03
	Muslim	1.43	0.78, 2.63	1.143	0.54, 2.43
Wealth index (Richest)	Poorest	1.26	0.86, 1.83	1.329	0.86, 2.05
	Poorer	1.13	0.77, 1.67	1.014	0.64, 1.60
	Middle	0.95	0.64, 1.42	1.019	0.64, 1.62
	Richer	1.08	0.73, 1.60	1.173	0.75, 1.84
Sex of child (Female)	Male	1.18	1.01, 1.37	1.163	0.98, 1.38
delivery place (Health)	Home	1.55	1.25, 1.91	1.555	1.22, 1.99
Baby postnatal (Yes)	No	0.75	0.57, 0.99	0.531	0.39, 0.73
Antenatal visits (4+)	0	0.97	0.78, 1.21	1.495	1.15, 1.95
	1–3	1.05	0.85, 1.29	1.372	1.07, 1.77
Mode of delivery (Caesarian)	Normal	0.10	0.06, 0.15	0.386	0.23, 0.64
Type of birth (Multiple birth)	Single	0.77	0.92, 0.53	0.597	0.33, 1.07
Size of child at birth (Small)	Large	0.94	0.77, 1.15	0.931	0.74, 1.17
	Average	0.80	0.66, 0.96	0.800	0.65, 0.99
Employment status (employed)	Not working	0.96	0.80, 1.15	0.836	0.68, 1.03
Parity (7+)	1–2	0.69	0.49, 0.97	0.847	0.58, 1.23
	3–4	0.85	0.66, 1.10	0.916	0.69, 1.22
	5–6	0.91	0.73, 1.15	0.893	0.69, 1.16

The region of residence of mothers was associated with early initiation of breastfeeding. The odds of early initiation of breastfeeding was lower among women residing in all regions except Harari compared to that of Dire Dawa (*p* - value < 0.01). Urban residents were 29% more likely to initiate breastfeeding early than mothers in the rural areas (OR 0.71, 95% CI 0.51, 0.99). The odds of delayed initiation of breastfeeding among Coptic orthodox was 1.89 (OR 1.89, 95% CI 1.02, 3.50) times higher than traditional/others. The male newborn was 1.18 times more likely to initiate breastfeeding late than a female (OR 1.18, 95% CI 1.01, 1.37). Regarding the place of delivery, mothers who delivered outside the health facility had higher odds of not initiating breastfeeding within 1 h of birth (OR 1.55, 95% CI 1.25, 1.91). The odds of delaying initiation of breastfeeding was lower among normal deliveries compared to cesarean birth (OR 0.10, 95% CI 0.06, 0.15), as were average size at birth compared to small birthweight (OR 0.80, 95% CI 0.66, 0.96) and newborn from small family size compared to those of seven and higher family size (OR 0.69, 95% CI 0.49, 0.97).

### Factors associated with exclusive breastfeeding

In multivariate logistic regression analysis, the independent variables region, mother’s age, father’s educational level, place of delivery, baby postnatal checkup, antenatal care during pregnancy, mode of delivery, and weight of the child at birth were significantly associated with exclusive breastfeeding. Mothers that lived in Affar (OR 0.17, 95% CI 0.10, 0.28), Somali (OR 0.16, 95% CI 0.09, 0.26), and Harari regions (OR 0.28, 95% CI 0.16, 0.49) were less likely to exclusively breastfeed compared to mothers living in the Dire Dawa region, whereas those from Tigray, Oromia, Benishangul-Gumuz regions and the Addis Ababa city had a higher odds of exclusive breastfeeding compared to Dire Dawa. A woman that had primary education was less likely to practice exclusive breastfeeding (OR 0.62, 95% CI 0.40, 0.96) compared to a secondary or higher education. Moreover, the odds of exclusive breastfeeding among babies born outside of health facilities, and mothers with no breastfeeding counseling during antenatal following up was lower (OR 1.56, 95% CI 1.22, 1.99) and (OR 1.50, 95% CI 1.15, 1.95), respectively, as compared to being born in health facilities and at least four antenatal follows up, respectively. Furthermore, a baby receiving a postnatal check (OR 0.53, 95% CI 0.39, 0.73) and children born of a medium sized at birth (OR 0.80, 95% CI 0.65, 0.99) were more likely to be exclusively breastfed (Table 2).

### Factors associated with duration of breastfeeding

From the multivariate Cox’s proportional hazard model analysis presented in Table 3, region, mother’s age, religion, wealth index, place of delivery, professional antenatal care, type of birth, baby weight at birth, employment status of mothers and parity were significantly associated with the duration of breastfeeding. Mothers who were living in Amhara (HR 1.31, 95% CI 1.05, 1.64), Oromia (HR 1.27, 95% CI 1.04, 1.54), and Benishangul-Gumuz (HR 1.34, 95% CI 1.09, 1.65) had a longer duration of breastfeeding than those who were residing in Dire Dawa.

**Table 3:** Factors associated with time to cessation of breastfeeding, Cox’s Proportional Hazard model, EDHS 2011 (*N* = 5122)

Background characteristics	Categories	Hazards ratio	95% CI) for hazards ratio
Region (Dire Dawa)	Tigray	1.15	0.92, 1.45
	Affar	0.99	0.80, 1.22
	Amhara	1.31	1.05, 1.64
	Oromiya	1.27	1.04, 1.54
	Somali	1.01	0.82, 1.24
	Benishangul-Gumuz	1.34	1.09, 1.65
	SNNPR	1.17	0.94, 1.45
	Gambela	0.96	0.75, 1.23
	Harari	1.03	0.82, 1.30
	Addis Ababa	1.06	0.81, 1.40
Place of Residence (urban)	Rural	1.03	0.88, 1.20
Maternal age (40 and higher)	≤ 24	3.24	2.68, 3.90
	25–29	2.30	1.96, 2.70
	30–34	1.94	1.67, 2.26
	35–39	1.45	1.25, 1.69
Husband education (Secondary / Higher)	No education	0.90	0.75, 1.09
	Primary	0.93	0.78, 1.11
Mother education (Higher)	No education	1.05	0.87, 1.26
	Primary	1.15	0.96, 1.39
	Secondary	0.97	0.80, 1.17
Religion (Traditional/Others)	Coptic orthodox	0.86	0.68, 1.09
	Protestant	0.82	0.65, 1.04
	Muslim	0.76	0.60, 0.96
Wealth index (Richest)	Poorest	1.27	1.08, 1.49
	Poorer	1.18	1.00, 1.39
	Middle	1.07	0.90, 1.26
	Richer	1.08	0.92, 1.27
Sex of child (Female)	Male	0.94	0.88, 1.01
Place of delivery (Health)	Home	0.77	0.70, 0.84
Baby postnatal checkup (Yes)	No	1.07	0.95, 1.22
Antenatal visits (4+)	0	0.90	0.81, 0.99
	1–3	1.04	0.94, 1.14
Mode of delivery (Caesarian)	Normal	0.85	0.67, 1.09
Type of birth (Multiple birth)	Single birth	1.39	1.04, 1.85
Size of child at birth (Small)	Large	0.80	0.73, 0.88
	Average	0.92	0.84, 0.99
Employment status (Employed)	Not working	1.26	1.16, 1.37
Parity (7+)	1–2	0.56	0.48, 0.66
	3–4	0.69	0.61, 0.78
	5–6	0.82	0.73, 0.92

Younger age mothers had a shorter duration of breastfeeding compared those 40 years and older. The average duration of breastfeeding were short (HR 0.76, 95% CI 0.60, 0.96) for mothers who were Muslims compared to that of traditional/others. Mothers who gave birth outside of health facilities had a shorter duration of breastfeeding compared to their counterparts who delivered in the health center. Furthermore, mothers who were not working, with poor wealth index and a single child had a longer duration of breastfeeding. Moreover, babies with a larger or average birthweight had a shorter duration of breastfeeding. Being born in a small family size also had a significant risk of early termination of breastfeeding (*p* - values < 0.001) (Table 3).

## Discussion

The present study set out to examine the factors associated with early initiation of breastfeeding, exclusive breastfeeding and duration of breastfeeding and trends in Ethiopia. The percentages of children who initiated breastfeeding in the first hour and exclusively breastfeed differs by region. Prior studies also reported only 42.20% babies born in Affar region were initiating breastfeeding within 1 h after birth [36] and only 50% of children in the Somali region was initiated breastfeeding early [37]. In contrast a cross-sectional study conducted in the Gozamin district [38] which reported 74.10% of the prevalence of the exclusive breastfeeding.

Initiating breastfeeding in the first hour was higher compared to a study conducted in Uganda [26], Tanzania [27] and Nigeria [28] which reported 56, 46.10, and 37%, respectively. The practice of exclusive breastfeeding was congruent with a similar previous study conducted in Uganda [26] which reported 46%, and in East Gojjam zone, Amhara regional state [29] reported 50.10%. In contrast, a study done in a Rajkot district of India 62% [30], Dilla Zuria District, Gedeo Zone 57.60% [31], Enderta woreda, of Tigray regional, state 70.20% [32], Ghana 64% [33], and Nigeria 56.60% [34] reported a higher proportion of exclusive breastfeeding. These differences might be the variation in health service utilization, culture, socioeconomic status of the study participants’, taboos about breastfeeding (colostrum) and methodological approaches used in the studies [39].

In the multivariate logistic regression analysis, it was found that mothers that lived in rural areas were less likely to initiate breastfeeding within 1 h of birth as compared to mothers that live in urban areas. This finding is consistent with previous studies conducted in Uganda [26], Nigeria [28], Tanzania [27, 40] and Ethiopia [41]. The possible explanation for this difference might be the higher percentage of rural women who delivered outside of health centers (i.e., home) without a health professional birth assistant. Additionally, home delivery and caesarean type birth were associated with delayed initiation of breastfeeding. The result is comparable with previous studies from Tanzania [27], Sri Lanka [42], Nigeria [28], India [43], Nepal [44], and a systematic review and meta-analysis of world literature [45]. This might be attributed to the fact that mothers who delivered into health centers could have a better chance of getting professional advice on feeding colostrum to her baby.

Mothers who had no education at all or only primary education were less likely to feed breast milk exclusively for the first 6 months. This finding is in agreement with previous studies reported in Ethiopia [46–48], Malaysia [49], and Nigeria [34, 50]. Maternal and child health care services such as postnatal and antenatal care, were risk factors of exclusive breastfeeding; i.e., mothers who received professional postnatal and antenatal care were more likely to exclusively breastfeed their infant. Several studies recognized that following health care services enable mothers to be aware of the benefits of exclusive breastfeeding to child and will get sufficient knowledge on nutrient content of breast milk enough for children during the first 6 months [51–53].

In this study, we observed that younger age mothers, Muslims, and employed women had terminated breastfeeding their baby at an earlier period. This finding is consistent with a study conducted in Kuwait [54] and Gondar town, Ethiopia [55]. The possible explanation of this might be that younger women are interested to have more children and employed mothers spend more of their time at the work place.

Children born of mothers that delivered at home and who are multiple births were more likely to terminate breastfeeding earlier [56]. Infants with an average or large birthweight were more likely to a short duration of breastfeeding.

### Strength of the study

It uses a nationally representative survey data set, which enhance inferences for the entire country level.

### Limitation of the study

This study is a retrospective report based on mother’s recall/perception of the events that took place for the past 5 years from the time of breastfeeding initiation to termination. The major limitations were it is subject to recall bias. The other weakness of the study was that some important possible factors that could affect the practice are missed due to incompleteness of information.

## Conclusions

The study has examined the factors that are associated with early initiation of breastfeeding, exclusiveness, and the duration of breastfeeding in Ethiopia, based on Ethiopian DHS 2016 data.

Regional differences, place of residence, place of delivery, professional antenatal and postnatal care, education of mother, employment status of mothers, birth type, age of mother, wealth index, religion, mode of delivery, size of a baby at birth and parity were significant independent variables associated with breastfeeding practices in Ethiopia. Suboptimal breastfeeding patterns still exist in Ethiopia. Providing health education and counseling for mothers during and after pregnancy in the study setting are important to encourage mothers to deliver at health centers and get professional counseling on early initiation and exclusive breastfeeding.

## Data Availability

The datasets used and/or analyzed during the current study are available from the corresponding author on reasonable request.

## Abbreviations

CSA: Central Statistics Agency
DHS: Demographic and Health Survey
EDHS: Ethiopia Demographic and Health Survey
HR: Hazard Ratio
LRT: Likelihood Ratio Test
MoH: Ministry of Health
NNP II: National Nutrition Programme II
OR: Odds Ratio
SDGs: Sustainable Development Goals
SSA: Sub-Saharan Africa
UNICEF: United Nations Children’s Fund
USAID: United States Agency for International Development
VIF: Variance Inflation Factor
WHO: World Health Organization
